# Supplementary figures and images for: A standardized quantitative analysis strategy for stable isotope probing metagenomics
Source: mSystems. 2023 Jun 28;8(4):e01280-22. doi: 10.1128/msystems.01280-22 (PMC10469821; doi:10.1128/msystems.01280-22)

Condition

- Condition A (4%)
- Condition B (8%)
- Condition C (11%)
- Condition D (31%)
- Condition E (11%)
- Condition F (22%)
- Condition G (32%)

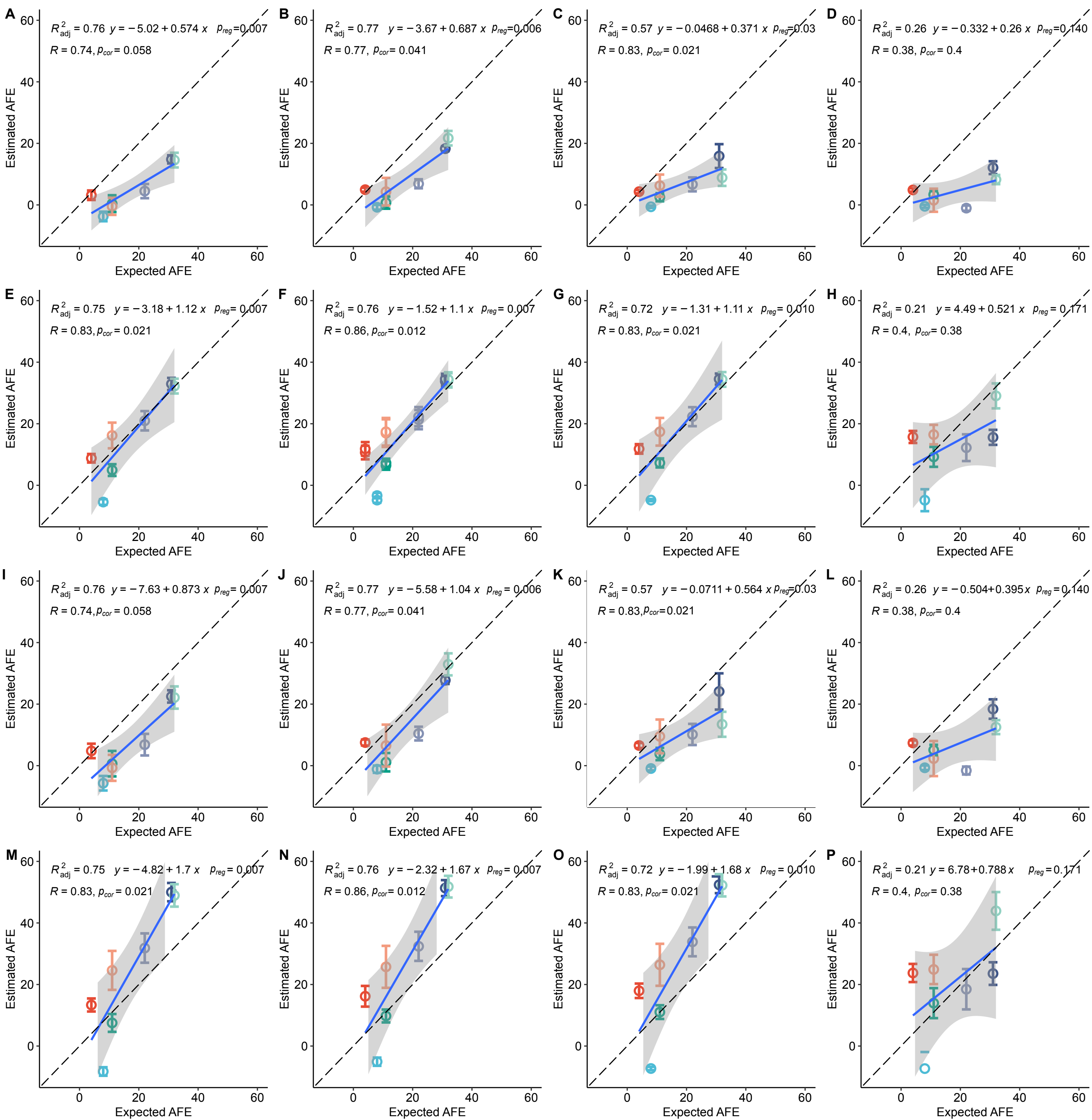

Supplement: Figure S3 — Linear regression parameters and Spearman correlations between estimated and expected AFEs obtained using the modified qSIP model (A-H) and the ΔBD method (I-P). (A and I) raw coverage, (B and J) relative coverage, (C and K) multiplying relative abundance with DNA concentration following Greenlon and colleagues (25), (D and L) multiplying relative coverage with DNA concentration following Starr and colleagues (24), (E and M) Sequin approach with ordinary least squares regression without Cook's distance filtering (F and N) Sequin approach with ordinary least squares regression with Cook's distance filtering (G and O) Sequin approach with robust linear regression, and (H and P) Relativizing abundances per fraction (MAG abundance/sum of MAG abundances in each fraction) from sequin approach with robust linear regression. preg and pcor correspond to the P-values for the linear regression and Spearman correlation. The intercepts determined by linear regression were not significantly different from zero (P-value > 0.05) in any method for estimating abundance. [file msystems.01280-22-s0003.pdf]

Condition

- Condition A (4%)
- Condition B (8%)
- Condition C (11%)
- Condition D (31%)
- Condition E (11%)
- Condition F (22%)
- Condition G (32%)

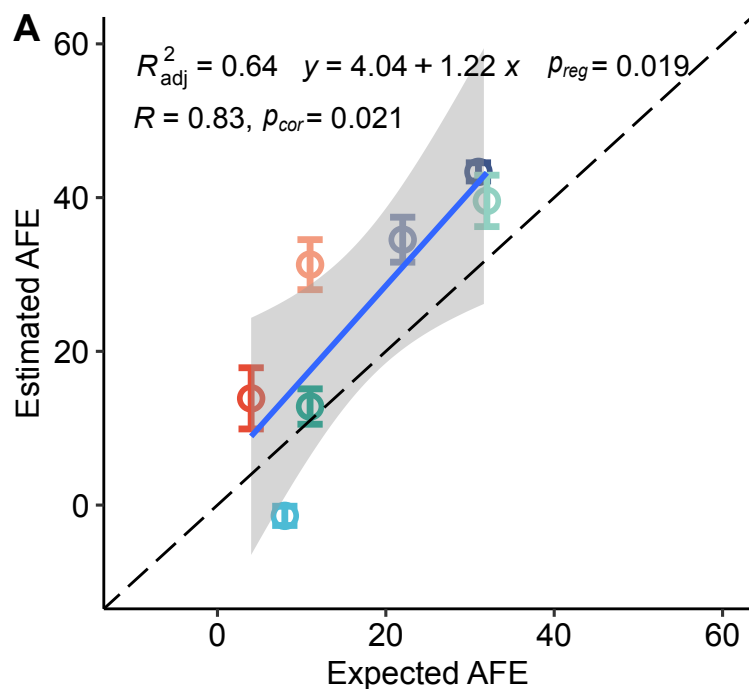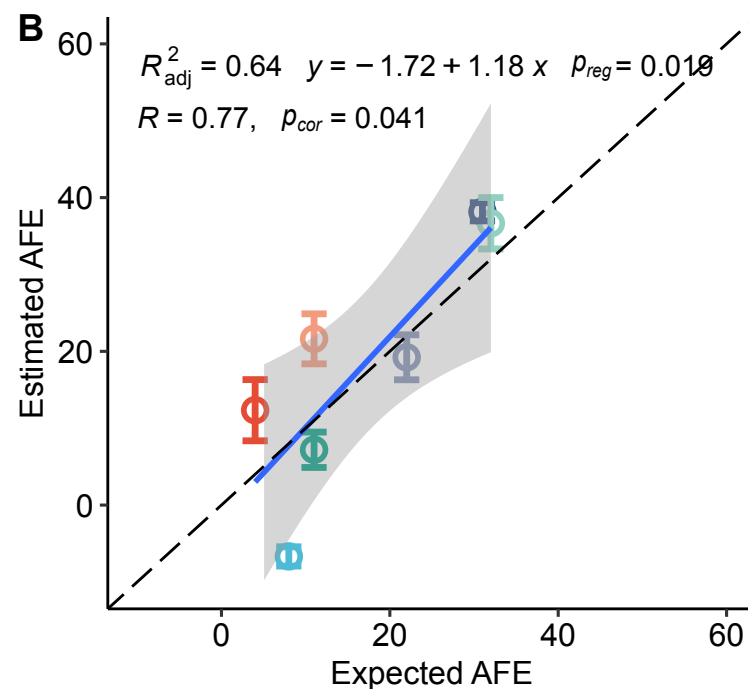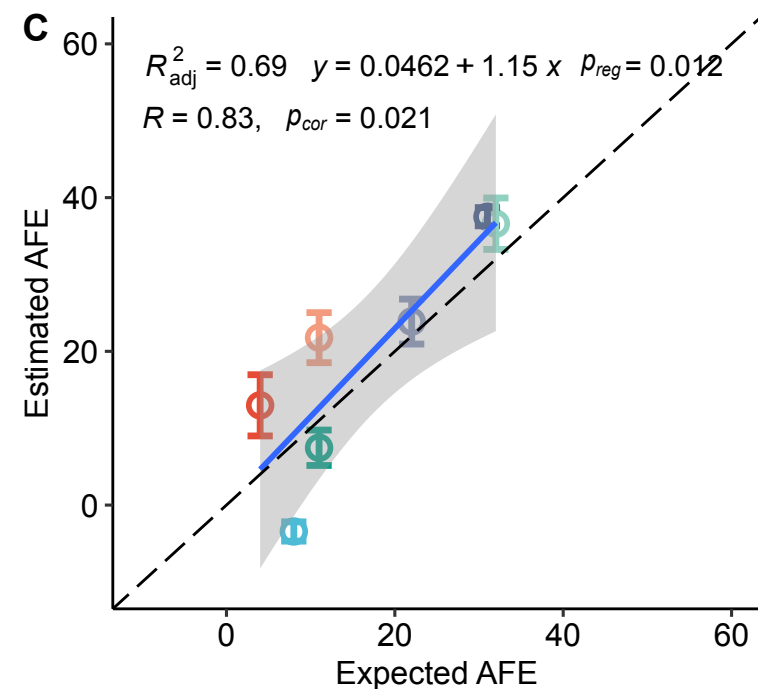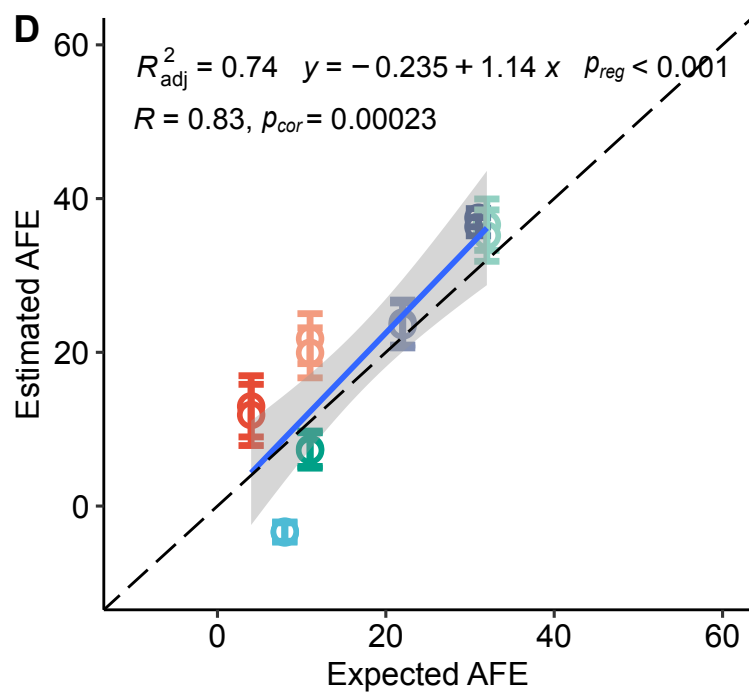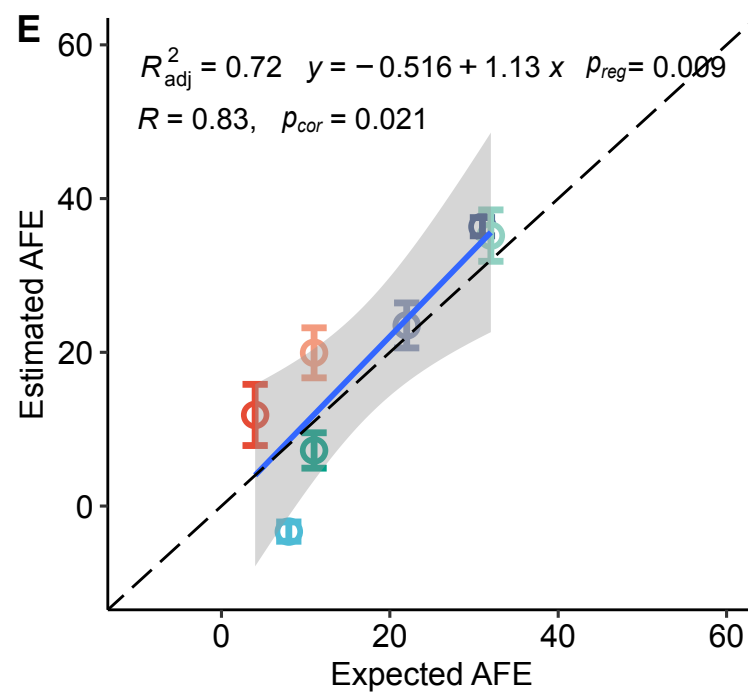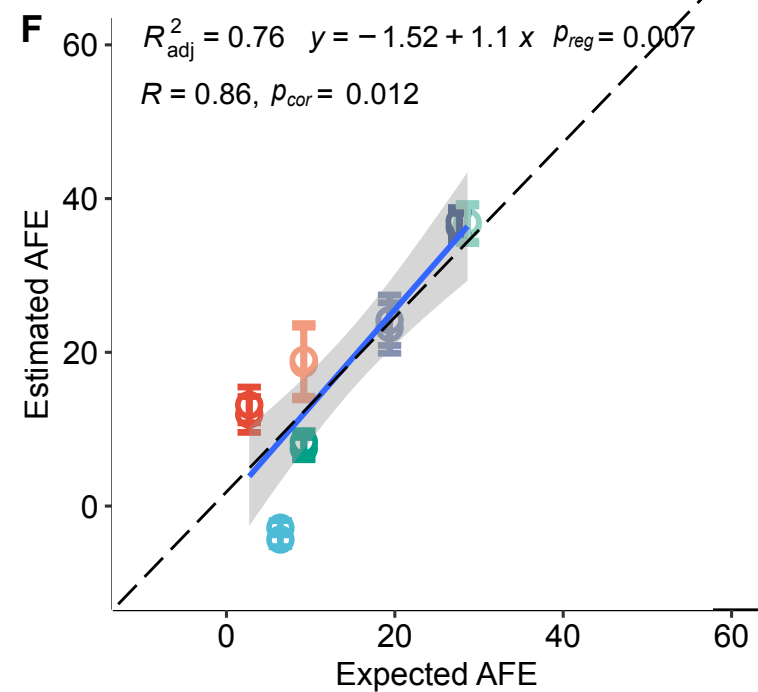

Supplement: Figure S4 — Linear regression parameters and Spearman correlations between estimated and expected AFEs obtained using the qSIP method for subsampled data at mean cumulative coverages of (A) 0.01X, (B) 0.1X, (C) 1X, (D) 10X, (E) 100X, and (F) 1000X. preg and pcor correspond to the P-values for the linear regression and Spearman correlation. The intercepts determined by linear regression were not significantly different from zero (P-value > 0.05) at any level of subsampling. [file msystems.01280-22-s0004.pdf]
