# Supplementary material for: A standardized quantitative analysis strategy for stable isotope probing metagenomics
Source: mSystems. 2023 Jun 28;8(4):e01280-22. doi: 10.1128/msystems.01280-22 (PMC10469821; doi:10.1128/msystems.01280-22)
Supplement: Supplemental Text — Additional methodological details regarding experimental design, sequencing, assembly, and data analysis. [file msystems.01280-22-s0009.docx]

**Supplementary Information**

**“A standardized quantitative analysis strategy for stable isotope probing metagenomics”**

Dariia Vyshenska, Pranav Sampara, Kanwar Sing, ​​Andy Tomatsu, W. Berkeley Kauffman, Erin E. Nuccio, Steven J. Blazewicz, Jennifer Pett-Ridge, Katherine B. Louie, Neha Varghese, Matthew Kellom, Alicia Clum, Robert Riley, Simon Roux, Emiley A. Eloe-Fadrosh, Ryan M. Ziels, Rex R. Malmstrom

**Read quality filtering**

Raw reads were quality filtered and trimmed with the RQCFilter2 software (<https://github.com/sghignone/BBTools>) using the following parameters: rqcfilter2.sh rna=f trimfragadapter=t qtrim=r trimq=0 maxns=3 maq=3 minlen=51 mlf=0.33 phix=t catdogmousehuman=t khist=t detectmicrobes=t sketch barcodefilter=f. We removed any replicate for which the average number of reads across all fractions was less than 5M.

**Metagenome assembly parameters**

QC filtered and trimmed reads were then used for generating assemblies. The following four strategies were used for contig assembly: a) an assembly of unfractionated SIP replicate sample with metaSPAdes; b) a single fraction assembly with metaSPAdes (371 assemblies); c) a single sample co-assembly with metaSPAdes(v3.15.2) (co-assembly of all fractions sequenced for a single SIP replicate sample, 24 assemblies); d) an experiment-wise co-assembly with MetaHipMer(v.2.0.1.2) (assembly of all fractions across all replicates).

The following workflows, software version, and parameters were used for assembling contigs:

1. MetaSPAdes pipeline

The assemblies were generated either from a single .fasta file (fraction assemblies, unfractionated SIP sample) or from a set of concatenated .fasta files (single SIP replicate sample co-assemblies). The following steps were performed to generate the assemblies:

Reads were corrected using bbcms with bbcms version "38.90". This was run using

the following command-line options: bbcms.sh -Xmx100g metadatafile=counts.metadata.json mincount=2 highcountfraction=0.6 in=bbcms.input.fastq.gz out1=input.corr.left.fastq.gz out2=input.corr.right.fastq.gz.

The readset was assembled using metaSPAdes assembler with metaspades version

"3.15.2". This was run using the following command line options: spades.py -m

2000 --tmp-dir cromwell_root -o spades3 --only-assembler -k 33,55,77,99,127 --meta -t 16 -1 input.corr.left.fastq.gz -2 input.corr.right.fastq.gz.

The input read set was mapped to the final assembly and coverage information generated with bbmap version "BBMap:38.86". For co-assemblies, each fasta file used in generating a co-assembly was mapped to the assembly.contig.fasta file separately. This was run using the following command line options: bbmap.sh build=1 overwrite=true fastareadlen=500 -Xmx100g threads=16 nodisk=true interleaved=true ambiguous=random rgid=filename in=reads.fastq.gz ref=reference.fasta out=pairedMapped.bam.

1. MetaHipMer pipeline

The assembly was generated from all generated .fasta files that passed all filtering criteria (including correct distribution of pre-centrifugation spike-ins, see details below). The following steps were performed to generate the assembly:

Filtered reads were co-assembled with MetaHipMer 2 version 2.0.1.2.593-gf6b15c2-master

[mhm2.py -k 21,33,55,77,99 --post-asm-align --post-asm-abd] on 1600 Haswell nodes on the NERSC Cori system. Contigs smaller than 500 bp were removed. Alignment information was determined by mapping reads to the assembly reference with BBtools version 38.95 [bbmap.sh nodisk=true interleaved=true ambiguous=random mappedonly=t trimreaddescriptions=t usemodulo=t fast=t]. Coverage was determined by running BBTools version 38.95 [pileup.sh].

**Metagenome binning and dereplication**

All assemblies were binned independently from each other. For co-assemblies, we used a set of separate coverage files (all fractions’ coverage files; for each co-assembly we used coverage of these fractions from which the assembly was re-created). We used the binning pipeline which is a part of the previously described DOE JGI Metagenome Workflow (58). Genome-wide Average Nucleotide Identity (gANI) and alignment fraction (AF) were calculated pair-wise for all medium and high-quality genome drafts (36). Prokka(v1.14.6) was used to generate .gff files for gANI and AF calculation (parameters: prokka --metagenome)(doi: 10.1093/bioinformatics/btu153). MAGs with a minimum pair-wise ANI >= 96.5 and AF >= 30 were assigned to the same cluster.

To select a single representative genome draft for each cluster, the following steps were performed one after the other: 1) if a cluster contained both medium- and high-quality MAGs, medium-quality MAGs were filtered out; 2) MAGs with completeness lower than truncated maximum completeness of the remaining MAGs were filtered out; 3) MAGs with contamination values equal minimum contamination value of the remaining MAGs were selected; 4) if more than one MAG was left, MAG with the largest total value was selected; 5) if, after application of steps 1-4, more than one MAG was left, we randomly selected one as a final representative MAG of the cluster.

After selecting a representative MAG for each cluster, we renamed each MAG’s scaffolds with a unique name. All spike-in sequences and representative MAG scaffolds were concatenated into a single file, and was used as a reference for a new round of fraction reads mapping with BBmap(v38.96) using these parameters: bbmap.sh nodisk=true interleaved=true ambiguous=random ref=reference.fa out=pairedMapped.bam covstats=covstats.txt bamscript=to_bam.sh.

**Quality filtering of fractions**

We checked each fraction if the total coverage for either pre-centrifugation spike-ins or sequins was more than zero. All fractions passed this test.

**Detection and removal of anomalous samples using pre-centrifugation spike-ins**

To test if any of the replicates was mishandled during sample processing, we performed the following analysis for each normalization tested in this research:

1. Normalized coverage data of pre-centrifugation spike-ins;
2. Removed 100% pre-centrifugation spike-ins data due to the fact that the expected density of that spike-in was outside of the detected DNA density range;
3. For each replicate and for each pre-centrifugation spike-in within that replicate we found densities of the maximum normalized coverage;
4. For each replicate order pre-centrifugation spike-ins based on maximum coverage density values calculated in step #3;
5. Compared the order of the spike-ins determined in step #4 to the expected order. The expected order of spike-ins was calculated based on the order of expected density (g/mL) per spike-in sequence: ED = 0.098 * GC + 1.66 + 0.036 * L

Where: ED - Expected Density (g/mL); GC - GC-ratio of the spike-in sequence (measured from zero to one); L - C^13^ label ratio (measured from zero to one) of the spike-in

1. If at least two pre-centrifugation spike-ins within one replicate had order mismatching with expected - the replicate was flagged as mishandled and removed from the following analysis.

We removed three replicates from the control condition sequenced in a separate batch and did not pass pre-centrifugation spike-in quality filtering criteria based on absolute or relativized absolute abundance data.

**Subsampling of *E. coli* reads**

We performed the following steps using samtools(v1.7) (htslib 1.7) to extract and subsample reads that mapped to *E. coli* MAG:

1. .bam files generated via mapping reads with BBmap against deduplicated MAG set were sorted using samtools sort command;
2. .bed file that contains information for all contigs of *E. coli* MAG was constructed;
3. *E. coli* reads from sorted .bam files were extracted:

*samtools view -b sorted.bam -L ecoli_mag.bed > ecoli-reads.bam*

1. Then, the extracted *E. coli* reads were subsampled (where, X is a unique seed; and, Y is one of the following values: '00001', '0001', '001', '01', '1'):

*samtools view -s X.Y -b ecoli-reads.bam > subsampled_data.sam*

1. .sam files were transformed into .bam using:

*samtools view -S -b subsampled_data.sam > subsampled_data.bam*

1. new coverage values based on subsampled data were calculated:

*pileup.sh in=subsampled_data.bam out=subsampled_data_cov.txt*

**Improving sequin addition by measuring sample DNA concentrations in SIP fractions**

Sequins should be added to SIP fractions prior to PEG precipitation and DNA resuspension steps in order to track variability introduced during DNA recovery, library creation, and sequencing. Sequin additions in our mock microbiome study were based on expected DNA concentrations within each fraction, but in future studies it would be helpful to target a specific ratio of sequins to sample DNA to ensure sufficient sequin coverage in cases where actual sample concentrations differ from expectations. For example, sequencing coverage of the most abundant sequin ranged from 73X to 2,043X in fractions from the unlabeled control replicates. This was sufficient for sequin-based quantification, but if sample DNA recovery had been substantially higher in some fractions, it is possible the depth of sequin coverage might have been too low for reliable quantification. Thus, adjusting sequin additions to match actual sample DNA concentrations measured before PEG precipitation would ensure sufficient coverage in future studies.

To tailor sequin additions to the amount of sample DNA in each fraction, we assessed the feasibility of measuring DNA directly from the SIP fractions before PEG precipitation and when DNA was still in concentrated CsCl. That is, we tested the impact of adding different volumes of CsCl ultracentrifugation gradient solution (1.884g/ml CsCl in Tris-EDTA + KCl) on the accuracy of DNA concentration measurements. We added 1uL of a 0.5ng/uL DNA and between 0-9uL of CsCl gradient solution to 200uL of Quant-IT DNA High Sensitivity Assay Kit reagents (ThermoFisher; Q33120). Sample volumes and DNA concentrations were held constant by adding H_2_O such that the sum of DNA, CsCl gradient solution, and H_2_O additions equaled 10uL. Fluorescence endpoint measurements (485ex/528em) of five replicates were collected with the Synergy H1 plate reader (Biotek) and compared to a standard curve per the Quant-IT standard 96-well plate protocol.

**
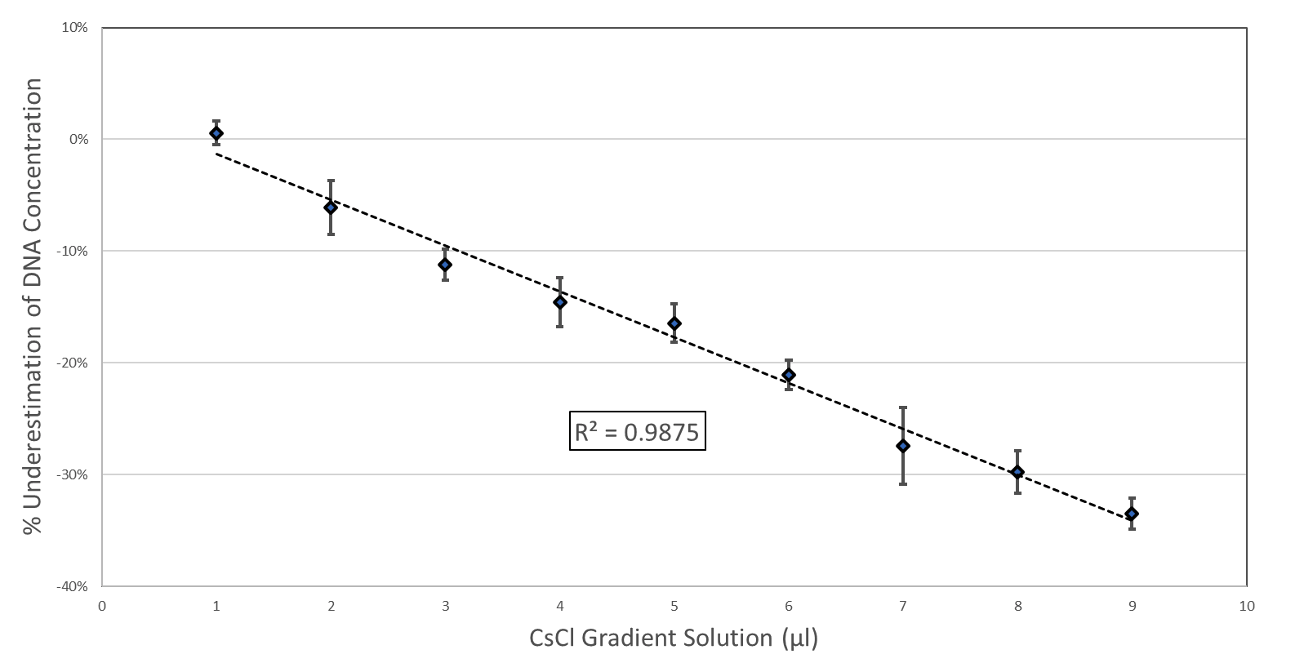
**

**Supplementary Information Figure 1:** Impact of SIP CsCl gradient solution on measurements of DNA concentrations made with the Quant-IT DNA High Sensitiviy Assay Kit. The error bars indicate standard deviation (n=5). The dashed line indicates a linear regression (R^2^=0.9875; F-test *p-value* = 6.32 X 10^-8^).

We observed a consistent linear decrease in the measured DNA concentration based on the amount of CsCl gradient solution in each sample (**Supplementary Information Figure 1**). DNA concentrations were underestimated by ~5% with a 2uL addition of gradient solution, and concentrations were underestimated by ~35% with a 9uL addition. This suggests the amount of sequins added to each fraction could be adjusted to sample DNA concentrations measured in SIP fractions containing high levels of CsCl. In addition, applying a correction factor based on the volume of CsCl gradient solution assayed could improve DNA concentration estimates. The impact of CsCl may differ depending on method used to determine DNA concentrations, and we recommend practitioners examine the impact of CsCl on their preferred assay methods.

***Pseudomonas putida* in sequence data files**

We initially intended to include ^13^C-labeled *Pseudomonas putida* KT2440 to the mock microbiome in addition to ^13^C-labeled E. coli, and labeled *P. putida* DNA was added to the various treatment conditions. Unfortunately, unlabeled *P. putida* DNA was accidentally omitted from the unlabeled control samples. Thus, there was no unlabeled baseline for measuring the isotopic enrichment of *P. putida* in treatment conditions, so it was excluded from subsequent analyses. *P. putida* reads remain in the fastq files of treatment conditions A-G.
